# AI-Driven Validation of Digital Agriculture Models

**DOI:** 10.3390/s23031187

**Published:** 2023-01-20

**Authors:** Eduardo Romero-Gainza, Christopher Stewart

**Affiliations:** Department of Computer Science and Engineering, The Ohio State University, Columbus, OH 43210, USA

**Keywords:** digital agriculture, decision tree, random forest, neural network, crop images, defoliation

## Abstract

Digital agriculture employs artificial intelligence (AI) to transform data collected in the field into actionable crop management. Effective digital agriculture models can detect problems early, reducing costs significantly. However, ineffective models can be counterproductive. Farmers often want to validate models by spot checking their fields before expending time and effort on recommended actions. However, in large fields, farmers can spot check too few areas, leading them to wrongly believe that ineffective models are effective. Model validation is especially difficult for models that use neural networks, an AI technology that normally assesses crops health accurately but makes inexplicable recommendations. We present a new approach that trains random forests, an AI modeling approach whose recommendations are easier to explain, to mimic neural network models. Then, using the random forest as an explainable white box, we can (1) gain knowledge about the neural network, (2) assess how well a test set represents possible inputs in a given field, (3) determine when and where a farmer should spot check their field for model validation, and (4) find input data that improve the test set. We tested our approach with data used to assess soybean defoliation. Using information from the four processes above, our approach can reduce spot checks by up to 94%.

## 1. Introduction

Digital agriculture is the fourth revolution of agriculture, wherein sensors, computation and artificial intelligence (AI) improve crop management, increasing yield. A common objective in digital agriculture is the early detection of signs of pest infestations. Unmanned Aerial Vehicles (UAVs) can fly over whole fields and capture images of crops [1,2,3]. Then, digital agriculture machine learning models can infer crop health from captured images. For example, state-of-the-art neural network [4] architectures can recognize defoliated leaves, a sign of infestation, appearing in aerial images [5]. Neural network models are increasingly common in digital agriculture because they can achieve greater classification accuracy than competing machine learning (ML) models [5]. However, the process that neural networks employ for classification involves complex, non-linear transformations on a multitude of variables and require large amounts of data inputs [6]. It is difficult to explain the behavior of neural network models, i.e., why the models label images the way they do. Other ML models, such as decision trees [7], use salient features within an image to make predictions. The behavior of these models is easier to explain. Decision trees can also achieve high accuracy in some cases, particularly when using bagging and boosting techniques to create ensemble models [8,9,10,11].

However, in digital agriculture, a finely tuned neural network still outperforms ensemble models [5]. When neural networks underlie digital agriculture models, farmers must trust the model without knowing how the model works. At best, farmers may see that the model performed well on other fields (test datasets), but they have little assurance that the model is working correctly on *their* field. This is particularly important when considering that many models are vulnerable to under and over-fitting and to biases in training and testing.

Ideally, farmers deciding to use a particular model in the field would be able to evaluate both the model and the datasets used to provide performance metrics of the model. Therefore, the farmer would want to:

1. Look at predictions made by the ML model and then go to the location of said prediction and evaluate its accuracy. This requires an understanding of how many images need to be observed to have a representative idea of model behavior, which in turn requires an understanding of how well a set of images covers all possible inputs in a field. Ideally, the images to be observed would be representative of the whole field while being as few as possible.
2. Assess how representative a test set is of a whole field. This is particularly important for identifying biases in a dataset. For instance, if all images on a field were taken at a certain time of day, maybe the model can learn to focus on a specific side of the image. A test set with the same bias would lead to high performance while the model would be likely to under-perform in practice. Results using a test set in which all features of the input are relevant are more likely to be representative of in-practice performance. Thus, measuring feature coverage can be an indication of the quality of a test set. Feature coverage also may be adjusted depending on the rigor needed by the farmer. A farmer could consider unnecessary that all features of a dataset are relevant and instead focus on the relevance of different regions of adjustable size. A model providing this representation assessment should adapt to different levels of quantization.
3. Add images to the test set, if necessary, so that the test set is more representative. This requires quantification of how representative is a dataset and how much one input contributes to the overall representation.
4. Evaluate the type of features of the input that make the model predict a certain result. While a perfect understanding might not be possible, an approximation is still useful for the farmer when making a decision. Using a separate interpretable model that learns from the original might lead to some additional knowledge on the original model.

We propose an approach that can achieve all four of these goals. Our approach starts by training a random forest [12] on the output of the neural network, i.e., the labels of the training set are the neural network prediction as opposed to ground truth. While this approach does not guarantee the random forest will output the same predictions as the neural network, it can provide hints on the neural network behavior. Then, we consider how the random forest uses the features available to its trees. Here features correspond to pixels or regions in the input images. We consider how many of the images of the test set are needed until all the features are used at least once. Expectations on how many images should be seen are given as lower and upper bounds by solutions of the coupon collector problem [13].

This approach allows farmers to know how many images must be seen until the model has considered all areas of all images, which gives an indication of how many images to see in the field so that the predictions of the model are evaluated in different scenarios. Further, understanding which images provide the most value can reduce the set of images to be observed. Simultaneously, the number of images needed in the test set to see all features indicates how well the test set represents all possible situations that could occur on the field. A test set in which more images are needed than those expected given the solution of the coupon collector problem probably is an unbalanced test set in which too many images share the same features. Additionally, an understanding of feature coverage given a set of images also provides information on what features are missing. Consequently, if a set of features is not present in a dataset, this approach indicates what features need to be present in additional images so that the feature coverage is complete. Finally, given a set of images, a random forest can indicate which features are relevant for the inputs [14,15,16]. While this is not a perfect correlation with feature importance on a neural network, if the random forest approximates the neural network closely enough, the features used by the forest might give insights on the behavior of the neural network.

We tested our approach using DefoNet [5] and a set of images taken by six UAV missions conducted in August and September of 2020 in five soybean fields in Wooster, Ohio, U.S. We ran DefoNet on the set of images and recorded its predictions. Then, we trained a random forest using DefoNet predictions as the class labels. We observed that our approach satisfied all goals described above while achieving a reduction of over 94% of the set of images that provide a full feature coverage with respect to the amount of images given by random selection.

## 2. Overview of System Proposed

**Figure 1:**
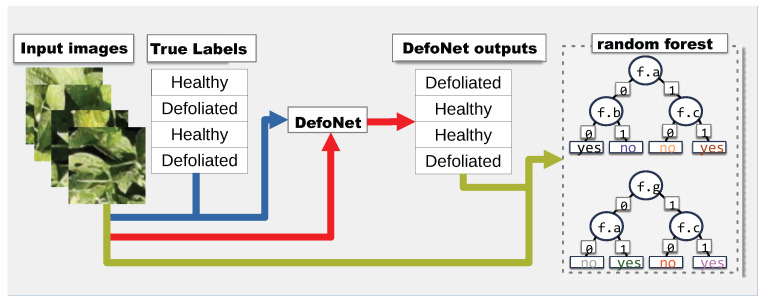
Overview of our approach. Images and the expert labels are used to train Defonet. Then, the same images are given to DefoNet to generate new labels. A random forest is trained with those images and DefoNet’s output.

Figure 1 gives an overview of our approach. First, images and expert generated labels are used to train a neural network (DefoNet in this case). Then, the same set of images are classified using DefoNet to produce another set of labels—the classifications of the neural network. The output of the neural network might differ from the expert generated labels, i.e., unless the neural network achieves 100% accuracy on the training set (which is typically undesirable as it signals over-fitting), the model will err on some inputs. Nonetheless, because the goal is for the random forest to learn the function of the neural network, the forest is then trained using the output of the neural network as labels, instead of the true labels. This produces a random forest trained to classify the output of the neural network. If the forest is trained effectively the forest will approximate the outputs of the neural network and the interpretation on forest classification could be indicative of what the neural network may be doing.

By following this technique, the four goals described above can be achieved. An interpretation of the neural network model can be obtained by running classification on a random forest. While there is no guarantee that feature importance will be the same in both models, the random forest can give a good indication of how the neural model works. A reasoning for this claim is provided in Section 3.4. This approach also allows for a method to quantify the coverage of a dataset, Section 3.5 discusses this method. The ability to add images to a dataset and to find a minimal set of locations to spot check are direct consequences of the method described in Section 3.5.

An added benefit of this approach is that the resulting random forest could potentially be used as a stand-alone model and outperform a random forest trained on ground-truth data. Decision trees are vulnerable to over-fitting due to noisy data. Since trees can split the input space indefinitely, they might learn data divisions that do not correspond to meaningful differences. While random forests are more robust, they still might under-perform if input data are inconsistent. However, the output of neural networks, due to their structure, must be a function in the strict mathematical sense, i.e., outputs of the neural network will be consistent. Thus, the training data that use the outputs of a neural network would not contain any noise. A random forest trained from noiseless data could potentially perform better on the test set predicting ground truth. However, this is a potentially beneficial consequence of our design, but not a part of the original design. Thus, for online predictions, we still rely on the neural network model and use the forest for the purposes described above.

## 3. Materials and Methods

### 3.1. Dataset

**Figure 2:**
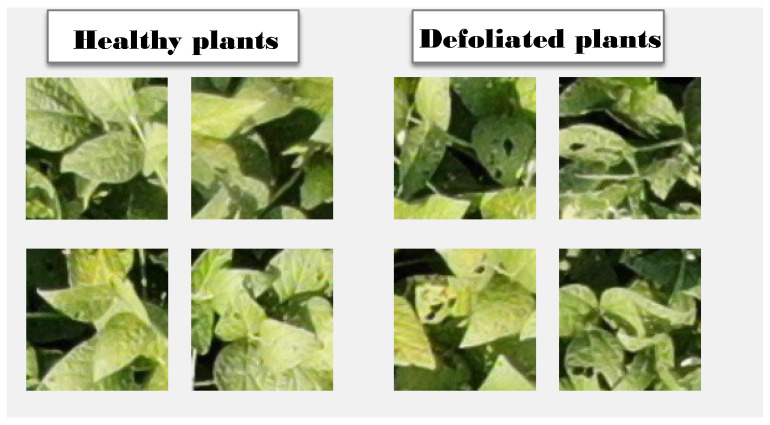
Example of images in the dataset. Images on the left are labeled healthy, while those on the right are labeled defoliated [5].

The images used for our experiment are the same images as the training set for DefoNet [5]. Figure 2 depicts some of the images of the dataset. The dataset consists of 97,235 images taken by six UAV missions conducted in August and September of 2020 in five soybean fields in Wooster, OH, USA. The images are all 108 × 108 pixels and are labeled healthy or defoliated if they show more than 10% defoliation. All images were expert-labeled. For training and testing the model, we separated all 97,235 images into a training set (with 87,182 images) and a test set (with 10,213 images). Both sets preserved the same proportion of positive (defoliated) and negative (healthy) samples.

### 3.2. Neural Network Model

**Figure 3:**
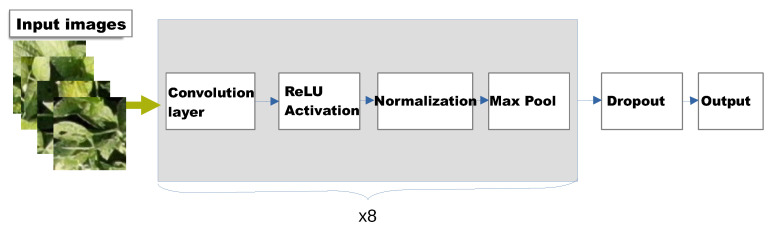
Architecture of DefoNet [5].

Neural networks [4] are popular ML models used for classification. They use one or more layers of functions to produce a classification output. If a neural network with multiple layers uses at least one convolution layer, then the network can be referred to as a convolutional neural network (CNN) [17]. CNNs perform better in computer vision tasks [17]. For our neural network, we used DefoNet [5] which is a fine-tuned CNN designed to perform well on defoliation recognition. Figure 3 illustrates the architecture of DefoNet. In Defonet, the inputs are images of 108×108 pixels. These inputs are then passed through 8 convolutional layers all with 3×3 filters. Layers have varying number of filters (from 32 to 128). After each convolutional layer, there is a ReLU activation function, followed by normalization and pooling layers. Finally, there is a dropout layer before a fully connected layer which gives the output.

### 3.3. Random Forest Training

**Figure 4:**
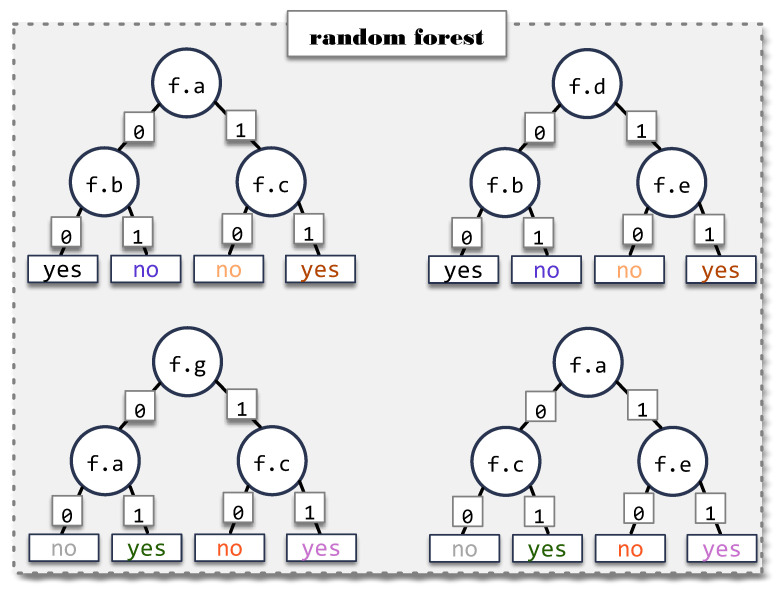
Example of a random forest.

Decision trees [7] are ML models that classify inputs by repeatedly separating input samples based on the values of certain features. Decision trees learn these splits by considering potential information gain splitting at each feature. While effective, this method is vulnerable to over-fitting and to noise in the input data. A random forest [12] is an ensemble of decision trees in which every tree has access to a different subset of samples and features during training. Figure 4 illustrates an example of a random forest with four decision trees. Features available to the trees are labeled f.a through f.g. Each tree uses a different set of features although they share some commonalities. This example uses binary classification (“yes” and “no”) although the structure can be generalized for more classes, trees and features. During testing, each tree in the forest contributes a vote (the classification of the tree) and results are aggregated to produce a classification for the forest.

We trained a random forest using Python Scikit-learn [18]—the most widely used framework for random forests—with 20 trees each with a maximum depth of 20. All other parameters were the default parameters. To train the random forest, we used the dataset described above but instead of the expert-generated labels, the labels given to the random forest were the outputs of DefoNet on the same set of images. The result is a random forest classifier trained to predict the behavior of DefoNet.

### 3.4. Characterization of a Neural Model through Random Forests

Neural networks use a combination of linear and non-linear layers to produce a classification [19,20]. This classification is a composite function on as many dimensions as the input has features. On the other hand, decision trees split the input field using lines perpendicular to the axis of each feature [21,22,23]. While this is a fundamental difference in how models operate, so is the difference between Riemann sums and definite integrals. Geometrically, both situations are analogous. Nonetheless, it is a well-known idea that Riemann sums can calculate the value of definite integrals [24]. Similarly, with sufficient input, a decision tree could exactly match the function produced by a neural network. Thus, others have considered before the idea of extracting information from a neural network into a decision tree [25,26,27]. However, in the case of Riemann sums, an infinite sum of infinitesimal areas is required for the value to match exactly that of a definite integral. Analogously, the amount of input needed to guarantee that the output of the decision tree exactly matches that of the neural network might be prohibitively large. For this reason, others have argued that the explanation of the neural model using a decision tree is not necessarily faithful [28]. While increasing input samples might not be feasible, increasing the number of trees can produce a better approximation as this also achieves more splits over the input space. Thus, a random forest could give a closer approximation of the function of a neural network. To be sure, random forests are harder to explain than individual decision trees. However, they maintain enough interpretability to provide a good idea on how the model works.

### 3.5. Quantification of Feature Coverage

ML models are vulnerable to learning particularities of their training set [29]. This is particularly dangerous when many samples in the training set have features in common. If the same features are relevant for classification in every sample of the training set, the model can learn to focus on those relevant features and ignore others. This is not a problem when other features are irrelevant in practice, however, if the ignored features are relevant in the inputs after the model is deployed, this can lead to decreased performance. Particularly, with images, models could learn to give higher importance to areas of the image that are key for classification. For aerial field images, certain areas of the input could have higher importance due to factors such as drone position, sun orientation, shadows, etc. For example, in a field that is mostly healthy but with a small defoliated area, drone images taken from similar positions would show the defoliated areas on the same side of the image, causing the model to ignore other groups of pixels.

This manifests differently in different kinds of ML models. In neural networks, understanding whether a feature is used or not might not be possible. Saliency maps [30] compute feature importance, but the outputs are real numbers. Low numbers could indicate that a feature is not used, but this is not precise nor guaranteed. On the other hand, random forests can directly and unequivocally output the features that are used to make a prediction.

**Figure 5:**
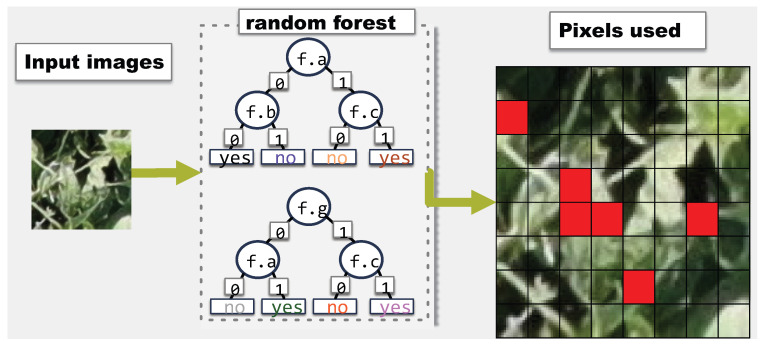
Example of feature coverage when considering individual pixels on an 8×8 image. Pixels in red are used for classification.

**Figure 6:**
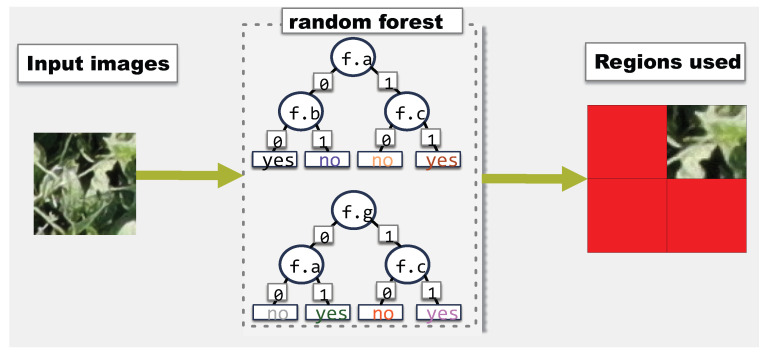
Example of feature coverage on the same input as Figure 5, but considering only whether any pixel in any of the four regions is used.

Figure 5 and Figure 6 show two examples of the feature usage output by a random forest. Figure 5 uses an 8×8 image instead of the 108×108 for clarity. In Figure 5, any time a pixel is used in any node of any of the trees, the pixel is marked as used. Pixels in red are marked as used. Figure 6 on the other hand, uses the same image as input, but here, the output does not have the same level of detail. Perhaps a farmer is only concerned about whether every region of the image is used for classification. In Figure 6, there are only four regions and the output is which of the four regions are used. Since the random forest is the same, the regions in Figure 6 correspond exactly with the location of the used pixels in Figure 5. Thus, the bottom left region is not used because no pixel from that area was used in the 8×8 image.

Ideally, models would make use of every feature available to them if the input requires it. In particular, in the case of an aerial image, every pixel has the same probability of being the location of defoliation. Thus, with a sufficiently extensive dataset, all features should be used at least once. This can be used as an indication of how representative a dataset is. However, depending on the rigor needed, measuring the usage of all pixels might be too much. Potentially, separating the input in regions would indicate the presence of shadows or the concentration of defoliation on the same area and require less input. Thus, the feature coverage should be adaptable to different levels of quantization. Further, evaluating feature coverage in the test set is more meaningful than in a training set. If a model uses all features in the test set, that means the training set was diverse enough for the model to not only use all features but learn their importance. We used a random forest to measure the feature usage of the model on the test set.

#### 3.5.1. Boundaries

Evaluating whether all features are used at least once might not be enough to assess a test set. If there is only one image that uses a certain feature, if that image is misclassified, accuracy could remain high. Thus, measuring feature usage against some expectation might be more informative.

An indication of how many images are required to use all the features in the input is given by the coupon collector problem [13]. This is a classic problem in which the collector randomly collects coupons until all *n* distinct types of coupons are collected. In this setting, the probability of collecting a certain coupon is constant, regardless of previously collected coupons. Therefore, some duplicate coupons are expected. The solution to the coupon collector problems gives an expectation of how many trials are required until all coupons are collected. If all coupons have the same probability of being collected, then the expected number of trials $\left(E\left[Y_{n}\right]\right)$ is:

(1) $$E\left[y_{n}\right]=n\sum_{i=1}^{n}\frac{1}{i}.$$

This is, however, fewer than the number of trials if the probabilities of every coupon are not identical due to increased probability of repetitions. In our approach, a trial consists of a visit to a node in any of the trees of the forest. A collected coupon corresponds to a previously unseen feature. Since decision trees are not structured to be random, features in our model do not have the same probability of appearing. For example, root nodes of every tree in the forest have a 100% probability of being collected in the first trial of a sample. Therefore, we take the previous result as a lower bound on the number of images that need to be seen.

Alternatively, instead of assuming every visited node on any sample has the same probability of giving a feature not previously used, we could assume that every image is slightly different than the rest and that each new image would use exactly one new feature. A dataset in which this was true would have very good feature coverage but it is unrealistic as images are likely to differ in more than one feature, and even if they do differ in exactly one feature that feature might not determine the classification of said input. However, as an average, this is a useful metric. If in a dataset all images on average added less than one feature, that would mean the dataset has too much repetition. Here, we can consider again the coupon collector problem. In this instance, coupons are still features, but a trial is a new sample image. Using the same formula as above, we have an upper bound for the number of images to be seen before all features are used.

#### 3.5.2. Application

The dataset we used consists of images that are 108×108 pixels. Thus, we had 11,664 features to track. Using our random forest, we measure how many images need to be classified before all 11,664 features are used at least once by the model. In other words, for all features *f*, we find a sample *s* such that while classifying *s*, some tree of our random forest splits the inputs based on the value of *f*.

Using the bounds defined in the previous section, we can compute the number of test samples required to use every feature. If the number is above the upper bound, this can indicate an unbalanced dataset. Further, this result can indicate to a farmer where to go on the field to evaluate the model predictions with a guarantee that the proposed set of images would provide full feature coverage, so, a full evaluation can be made. To be sure, the number of images to be visited might be very large. This can be reduced by relaxing the constraint on the model using every pixel and replacing it with using a pixel in every region. This allows for the definition of arbitrarily small regions and finding a set of images in which every region is relevant. This method can significantly reduce the number of locations on the field to visit and provides a tunable parameter for rigor.

Finally, this method can identify which features are less common in the dataset. If the dataset was judged to not cover the field well, then using the proposed approach, we can identify which of the characteristics of images that need to be added so that the dataset becomes representative.

### 3.6. Producing a Minimal Observation Set

Since random forest can highlight the set of features used in each sample, when finding the number of samples that have to be visited so that all features are used, our approach can also identify those images that contribute the most to coverage and remove redundancies. Thus, in our experiments, we first randomly selected images until all of the features were used and then pruned the set of selected images so that when used by the farmer to make observations on the field, the locations the farmer must visit are reduced.

## 4. Results

**Figure 7:**
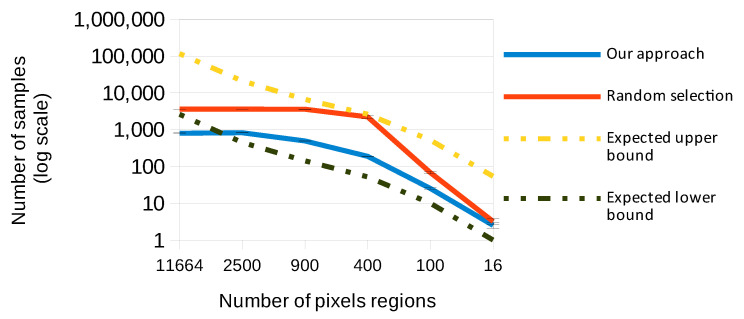
Number of samples needed until all features were used.

Figure 7 shows the average values over 10 runs of randomly selecting images until all features are used and of the minimal dataset obtained from pruning the random dataset using information given by the forest’s nodes. Both values are compared to the expected bounds given by the coupon collector problem. All the measurements were performed in our trained random forest using the test set described above. As described before, our model can adjust from finding all the features to finding if any feature in a region is used. This allows a farmer to choose how many locations on the field to observe, while balancing this decision with how much confidence they should have in the model. Figure 7 presents the number of regions of the image as the x-axis and shows the samples as the y-axis. The x-axis shows the total number of features counted individually, i.e., an image of 108 × 108 pixels has 11,664 features and we only consider 20 × 20 regions, that gives 400 features. Figure 7 also shows four different data points for every number of regions. The first line (blue) is the number of samples required to achieve full feature coverage using our method of finding the minimal set of images such that all features are used. The red line is the number of images needed to achieve full coverage when randomly selecting images, i.e., the blue line shows the value of the red one, but after the optimization. Black is the expected lower bound given by the solution of the coupon collector problem assuming identical probabilities for all features. Yellow is the expected upper bound, given by the solution to the coupon collector problem, assuming that every image provides only one new feature.

Noticeably, the expected upper bound in the samples with more pixels exceeds the number of available images in the test set (10,213). This is expected since the solution to the coupon collector problem does not factor in the number of samples available. Further, the two lines corresponding to measurements seem to exhibit asymptotic behavior. For larger sets of features, the results seem to approach the number of images available (namely, images classified as defoliated) in the test set for the random selection. For the minimal dataset, the result also seems to approach 800 when the set of features is large, but the requirements do not increase further as features increase. Both these results seem to indicate that there are some features that are uncommon in the dataset, but that slightly under 10% of the whole dataset is enough to cover all features.

**Figure 8:**
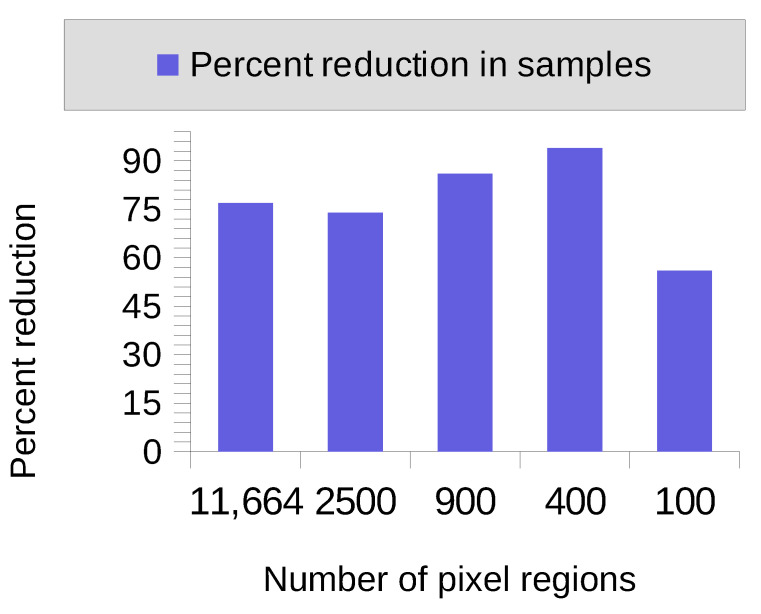
Percent decrease in samples needed to achieve full feature coverage by minimal set with respect to random sampling.

Figure 8 shows the decrease in the number of samples required to achieve full feature coverage by different levels of quantization (different number of pixels/regions). The case with 16 regions is not included in Figure 8 because both results are 2 images, so there is no reduction. For others, the results oscillate between 56% and 94%.

In terms of the accuracy of the model, the neural network still outperforms the random forest. However, our approach does not intend to replace the neural network model, but to complement it by adding interpretability and providing information for the farmer to make an informed decision on trusting the model. Thus, the accuracy of the model is the reported by DefoNet, i.e., over 90% accuracy and over 90% recall and precision [5].

## 5. Discussion

Our approach effectively trains a random forest to attempt to learn the function produced by a neural network. As discussed previously, this allows us to obtain certain insights from the model. This is not a result coming from an experiments, but a consequence of the used structures. Further, since neural networks are hard to explain, it is hard to effectively verify that the conclusions about DefoNet drawn through our random forest are correct.

In regards to our goal of evaluating whether a dataset is a good representation of the field, we observed on most inputs that the number of samples needed was between the expected upper and lower bounds, which indicates a good level of coverage. However, when using all the features available, the expected lower bound was higher than the number of images classified as defoliated, so the actual results were under the lower bound. While surprising, this fact makes sense given the formula used to solve the coupon collector problem. It is likely insightful that the dataset had a full feature coverage when using all of the images. However, future work might look into biased datasets and check how they compare. An additional detail to consider is how for inputs of size 20x20, the expected upper bound came very close to the actual measured value for random selection. This means that when evaluating a dataset for balance, the input size matters as specific input sizes might be outliers. For all experiments with more than 400 pixels, the number of samples needed in random selection was very close to the number of defoliated samples in the dataset. Future work might explore the point at which this starts to happen. It makes sense that for a sufficiently large number of features all samples are needed, but how many features are needed for this to occur might be insightful. Regardless, in all cases, the minimal dataset was much smaller than the random sampling. Thus, a few different levels of quantization are recommended so that outliers in the results can be more easily spotted. Nonetheless, the results in that case were still under the limit and the minimal set was far from the upper bound, which also supports the idea of a well-balanced set.

Finally, the results show that the minimal set of samples is consistently smaller than the set of samples given by random selection. This shows that the information available due to the interpretability of random forests helps reduce the set of samples a farmer would need to look at by up to 94%, thus showing the value of this technique to farmers.

## 6. Conclusions

Farmers can benefit from ML models that help with the timely identification of pests and other threats to crops. One type of such ML models are machine vision models that recognize defoliation in crops. Typically, these models use neural networks to maximize accuracy. However, neural network models are not interpretable. Thus, farmers may not have a way to verify that the model given is effective. While showing a high accuracy in a test set can induce some confidence, this represents performance of the ML model in another field. To trust the model given, a farmer might want to verify the model and the test set. For this, a farmer would want to:

1. Spot check specific locations in the field and compare to model prediction;
2. Evaluate the coverage of a dataset (test set) in a field;
3. Modify the dataset by adding images if coverage is not complete;
4. Understand why the model makes the predictions that it does.

We propose an approach in which the output of a trained neural network is used to train a random forest. We then show that this method can satisfy the first three goals described above and provide an approximation for the fourth one. We start by quantifying coverage of a field by comparing the usage of features with solutions to the coupon collector problem. Then, we find a set of images so that the coverage is complete. The quantification of coverage gives the evaluation of the dataset and the ability to recognize missing images in the dataset. The set of images with full coverage provides the list of locations to spot check. Finally, the random forest provides an approximation to an interpretation of the neural network. By employing this method, we showed that we can produce a set of locations to spot check that is 94% smaller than a randomly selected set that also has full coverage.

## Data Availability

Data and code available at https://github.com/EduardoRomero83/TreeExplorationOfDefoNet (accessed on 31 October 2022).
